# A randomized controlled crossover trial evaluating differential responses to antihypertensive drugs (used as mono- or dual therapy) on the basis of ethnicity: The comparIsoN oF Optimal Hypertension RegiMens; part of the Ancestry Informative Markers in HYpertension program—AIM-HY INFORM trial

**DOI:** 10.1016/j.ahj.2018.05.006

**Published:** 2018-10

**Authors:** Omar Mukhtar, Joseph Cheriyan, John R. Cockcroft, David Collier, James M. Coulson, Indranil Dasgupta, Luca Faconti, Mark Glover, Anthony M. Heagerty, Teck K. Khong, Gregory Y.H. Lip, Adrian P. Mander, Mellone N. Marchong, Una Martin, Barry J. McDonnell, Carmel M. McEniery, Sandosh Padmanabhan, Manish Saxena, Peter J. Sever, Julian I. Shiel, Julie Wych, Phil J. Chowienczyk, Ian B. Wilkinson

**Affiliations:** a Experimental Medicine & Immunotherapeutics Division, Department of Medicine, University of Cambridge, Cambridge, United Kingdom; b Experimental Medicine & Immunotherapeutics Division, Department of Medicine, University of Cambridge, and Cambridge, and Clinical Trials Unit, Cambridge University Hospitals NHS Foundation Trust, Cambridge, United Kingdom; c Department of Cardiology, Columbia University Medical Center, New York; d William Harvey Research Institute, Barts and the London School of Medicine and Dentistry, London, United Kingdom; e School of Medicine, Cardiff University, Heath Park Campus, Cardiff, United Kingdom; f Department of Renal Medicine, Heartlands Hospital, Birmingham, United Kingdom; g Department of Clinical Pharmacology, King's College London, British Heart Foundation Centre, London, United Kingdom; h Division of Therapeutics and Molecular Medicine, University of Nottingham, and NIHR Nottingham Biomedical Research Centre, Nottingham, United Kingdom; i Division of Cardiovascular Sciences, University of Manchester, Manchester, United Kingdom; j Blood Pressure Unit, Cardiology Clinical Academic Group, St George's University of London, Cranmer Terrace, London, United Kingdom; k Institute of Cardiovascular Sciences, University of Birmingham, Birmingham, United Kingdom; l Medical Research Council Biostatistics Unit, University of Cambridge, Cambridge, United Kingdom; m Office for Translational Research, Cambridge University Health Partners and University of Cambridge, Cambridge, United Kingdom; n Institute of Clinical Sciences, University of Birmingham, Birmingham, United Kingdom; o Department of Biomedical Sciences, Cardiff Metropolitan University, Cardiff, United Kingdom; p Institute of Cardiovascular and Medical Sciences, College of Medical, Veterinary and Life Sciences, University of Glasgow, Glasgow, United Kingdom; q Faculty of Medicine, National Heart & Lung Institute, Imperial College London, London, United Kingdom; r Experimental Medicine & Immunotherapeutics Division, Department of Medicine, University of Cambridge, and Cambridge Clinical Trials Unit, Cambridge University Hospitals NHS Foundation Trust, Cambridge, United Kingdom

## Abstract

**Background:**

Ethnicity, along with a variety of genetic and environmental factors, is thought to influence the efficacy of antihypertensive therapies. Current UK guidelines use a “black versus white” approach; in doing so, they ignore the United Kingdom's largest ethnic minority: Asians from South Asia.

**Study design:**

The primary purpose of the AIM-HY INFORM trial is to identify potential differences in response to antihypertensive drugs used as mono- or dual therapy on the basis of self-defined ethnicity. A multicenter, prospective, open-label, randomized study with 2 parallel, independent trial arms (mono- and dual therapy), AIM-HY INFORM plans to enroll a total of 1,320 patients from across the United Kingdom. Those receiving monotherapy (n = 660) will enter a 3-treatment (amlodipine 10 mg od; lisinopril 20 mg od; chlorthalidone 25 mg od), 3-period crossover, lasting 24 weeks, whereas those receiving dual therapy (n = 660) will enter a 4-treatment (amlodipine 5 mg od and lisinopril 20 mg od; amlodipine 5 mg od and chlorthalidone 25 mg od; lisinopril 20 mg od and chlorthalidone 25 mg od; amiloride 10 mg od and chlorthalidone 25 mg od), 4-period crossover, lasting 32 weeks. Equal numbers of 3 ethnic groups (white, black/black British, and Asian/Asian British) will ultimately be recruited to each of the trial arms (ie, 220 participants per ethnic group per arm). Seated, automated, unattended, office, systolic blood pressure measured 8 weeks after each treatment period begins will serve as the primary outcome measure.

**Conclusion:**

AIM-HY INFORM is a prospective, open-label, randomized trial which aims to evaluate first- and second-line antihypertensive therapies for multiethnic populations.

Hypertension is the single biggest contributor to the global burden of disease, a burden that is particularly great in lower- and middle-income countries.^1^ In high-income economies, ethnic minorities—often originating from lower- and middle-income countries—also appear to be disproportionately affected when compared to indigenous populations.2., 3., 4. Complex interactions between genes and the environment are thought to influence the pathophysiology of essential hypertension, the frequency of hypertension-related complications, and the response to treatment.^1^ However, data relating to ethnicity are complicated by the plethora of methods used to define *ethnicity* or *race*, and a greater understanding of environmental influences has led to the recognition that data collected in one country may not be readily applicable to similar ethnic groups in distinct geographical locations.^1^

European guidelines relating to the management of arterial hypertension make no allowance for ethnicity.^5^ In contrast, the North American guideline, published by the Joint National Committee in 2014, does, stating, “In the general black population, including those with diabetes, *initial* antihypertensive treatment *should include* a thiazide-type diuretic or CCB [calcium channel blocker].”^6^ Stratified by age and self-defined ethnicity (SDE), the UK's National Institute for Health and Care Excellence (NICE) recommends a third approach, with distinct initial monotherapies recommended for all those aged 55 years and over, as well as for younger black adults when compared to whites.^7^ However, the guideline makes no reference to South Asians (ie, those originating from the Indian subcontinent)—despite the fact that they represent the largest ethnic minority group in the United Kingdom at 4.7 million people (52.5 million “white British” citizens being the largest group within a total population of 65.6 million people).7., 8. Furthermore, the aforementioned guidelines fail to extend stratification to combination therapy.5., 6., 7.

Stratification on the basis of SDE is potentially flawed by virtue of an increasingly “admixed” population, the complex relationship between ethnicity and phenotype, and its inherent cohort-based approach which fails to account for interindividual variations.^9^ An alternative method of stratification seeks to use ancestry informative markers (AIMs)—genetic polymorphisms occurring with substantially different frequencies across populations from distinct geographical regions. Able to predict geographical ancestry, AIMs may capture the genetic component responsible for variations in drug response among ethnically diverse populations more discerningly than SDE.9., 10. Concurrent metabolomic profiling of plasma and urine (measurement of low– and intermediate–molecular weight metabolites which reflect the complex interplay between genetic, physiological, pathophysiological, and/or environmental factors) offers the potential to augment AIMs, with differences between individuals reflecting the entire spectrum of influences, especially diet.11., 12.

In an effort to address these issues, the AIM-HY INFORM trial intends to compare variations in response to antihypertensive agents among 3 cohorts of the UK population stratified on the basis of SDE, while also relating any variations to AIMs and metabolomic profiles. In doing so, we hope to evaluate the validity of current NICE guidance which has SDE at the center of its approach to pharmacotherapy and to examine whether use of AIMs and/or metabolomic profiling results in the more effective personalization of antihypertensive treatment. Furthermore, the trial will evaluate the efficacy of both monotherapy and dual therapy across all 3 cohorts and try to elucidate potential mechanisms underlying any difference in outcomes achieved by using SDE and AIMs. Thus, AIM-HY INFORM will enable clinicians to optimize their choice of antihypertensive treatments from current, generic, first- and second-line agents, reducing the attrition of antihypertensive therapies.

## Hypotheses

We hypothesize that the response to antihypertensive drugs (used either as mono- or dual therapy) differs by ethnicity.

Our secondary hypothesis relates to the possibility that AIMs and metabolites, and/or baseline hemodynamic measurements, are able to predict response to antihypertensive therapy.

## Methods

### Study design and objectives

AIM-HY INFORM is a multicenter, prospective, open-label study with 2 parallel, independent trial arms (mono- and dual therapy). Eleven UK sites will enroll a total of 1,320 patients. Those receiving monotherapy (n = 660) will enter a 3-treatment, 3-period crossover, lasting 24 weeks, whereas those receiving dual therapy (n = 660) will enter a 4-treatment, 4-period crossover, lasting 32 weeks. Equal numbers of all 3 ethnic groups (white, black/black British, and Asian/Asian British) will ultimately be recruited to each of the trial arms (ie, 220 participants per ethnic group per arm).

The primary objective of the AIM-HY INFORM trial is to determine whether the response to antihypertensive drugs differs on the basis of SDE. Secondary objectives (Table I) include an evaluation of this response on the basis of AIMs, baseline metabolomics, baseline hemodynamic data, genomics, and a more detailed evaluation of SDE (with a family tree extending to 3 generations, ie, grandparents). Additionally, the trial aims to determine (1) the most effective mono- and dual therapy for hypertension and any variation(s) by ethnicity, (2) whether metabolomics and hemodynamics differ by ethnicity, and (3) whether previously identified biomarkers (ie, those derived from other cohorts, eg, the United States) can predict the therapeutic response observed. Further exploratory, tertiary objectives may be defined.

**Table I:** Trial objectives

Primary objective
- To determine whether the response to antihypertensive drugs differs on the basis of SDE
Secondary objectives
- To determine if the response to antihypertensive drugs differs by:
- AIMs
- Baseline metabolomics
- Baseline hemodynamics
- Genomics
- Detailed SDE (family tree extending to grandparents)
- To compare detailed SDE with AIMs as a cause for the response to antihypertensive drugs
- To determine the most effective mono- and dual therapy for hypertension and whether this varies by ethnicity
- To determine whether metabolomics and hemodynamics differ by ethnicity
- To test whether previously identified biomarkers (derived from other cohorts, eg, United States) can predict drug response

### Study population, treatment assignment, and randomization

The inclusion and exclusion criteria are listed in Table II. Hypertensive adults aged between 18 and 65 years are eligible for inclusion provided that they are able to self-identify with 1 of the 3 ethnicities outlined. Treatment-naive individuals will be confined to the monotherapy arm. Those who have previously been treated/are being treated with antihypertensive agents will be able to enter either arm provided that they are able to undergo a washout of 2-4 weeks; if not, they will be assigned to dual therapy.

**Table II:** Selection criteria

Inclusion criteria
1. Able to give written informed consent
2. Aged 18 to 65 y
3. SDE falling into 1 of 3 groups:
- White (white British, white Irish, or any other white background)
- Black/black British (black Caribbean, black African, or any other black background)
- Asian/Asian British (Asian Indian, Asian Pakistani, Asian Bangladeshi, or any other South Asian background)
4. Hypertensive as defined by:
- *Monotherapy*
- Treatment-naive patients:
Daytime average SBP ≥135 mm Hg or DBP ≥85 mm Hg
Using ambulatory blood pressure monitoring (ABPM) or home blood pressure monitoring (HBPM) (validated device based on an average of 10 readings)
- Treated patients:
Daytime average SBP ≥135 mm Hg or DBP ≥85 mm Hg
Using ABPM or HBPM (validated device based on an average of 10 readings), if: Likely to achieve control on a study drug while being willing **and** able to complete 2-4 wk washout
- *Dual therapy*
- Treated (with 1–3 antihypertensive agents):
Daytime average SBP 135-200 mm Hg and/or DBP 85-110 mm Hg
Using ABPM or HBPM (validated device based on an average of 10 readings)
Exclusion criteria
a. Inability to identify with one of the predefined ethnic groups, eg, admixed origin
b. Pregnant or breastfeeding women
c. Known or suspected secondary hypertension
d. Significant sensitivity or contraindications to study medicines
e. Concomitant lithium or variable-dose non-steroidal anti-inflammatory drugs (NSAID) use
f. A requirement to take any of the study drugs continuously, eg, ACEi and heart failure
g. Clinically significant hepatic impairment
h. Clinically significant kidney impairment
i. Concurrent clinical trial participation (systemically vasoactive medicines or drugs known to interact with the study medicines)
j. Patients deemed unsuitable by the investigator on clinical grounds, eg, patients in atrial fibrillation

SDE: self defined ethnicity; SBP: systolic blood pressure; DBP: diastolic blood pressure; NSAID: non-steroidal anti-inflammatory drugs; ACEi: angiotensin converting enzyme inhibitors.

Following allocation to a trial arm, subjects will be randomized to a sequence of drugs (Figure) using an online system (https://www.sealedenvelope.com). This requires the participant screening number, initials, date of birth, and SDE, along with the site name, confirmation of eligibility, and trial arm allocation. For those receiving monotherapy, 6 possible treatment sequences may be generated, as the intervention consists of a 3-treatment, 3-period crossover: ABC, ACB, BAC, BCA, CAB, and CBA. Where subjects are allocated dual therapy (a 4-treatment, 4-period crossover), 4 possible treatment sequences exist: ABDC, BCAD, CDBA, and DACB. In both instances, a Latin square balanced for first-order carryover effects is deployed^13^; the randomization schedule will also aim to have equal numbers of participants in each sequence.

**Figure:**
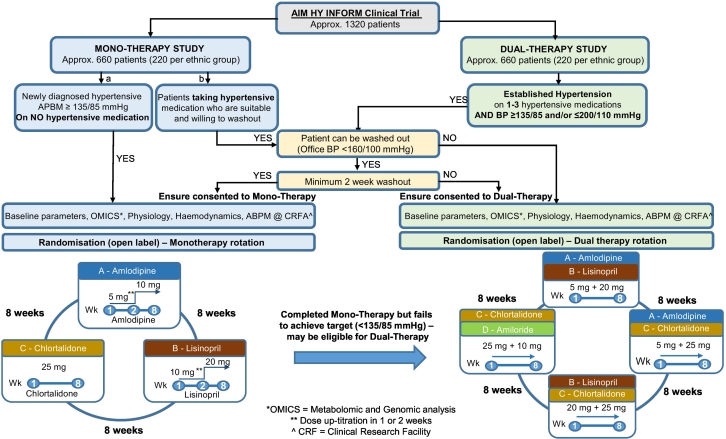
Trial flowchart.

Prescribed in an open-label manner, the treatments allocated to each letter are detailed in Table III. Stable concomitant therapy for unrelated indications is permitted provided that it does not have an antihypertensive effect; dose modifications for the trial drugs are not permitted. As this is a pragmatic trial of licensed medications, overall adherence will be assessed by urine drug screening for the study medications in a randomly selected subgroup of individuals (n = 50). Participants will also be asked to self-report on compliance; where this falls below 75%, subjects may be withdrawn at the discretion of the principal/chief investigator(s).

**Table III:** Selection criteria

Monotherapy
A. 1-2 wk of amlodipine 5 mg od followed by 6-7 wk of amlodipine 10 mg od
B. 1-2 wk of lisinopril 10 mg od followed by 6-7 weeks of lisinopril 20 mg od
C. Approximately 8 wk of chlorthalidone 25 mg od
Dual therapy
A. Approximately 8 wk of amlodipine 5 mg od and lisinopril 20 mg od
B. Approximately 8 wk of amlodipine 5 mg od and chlorthalidone 25 mg od
C. Approximately 8 wk of lisinopril 20 mg od and chlorthalidone 25 mg od
D. Approximately 8 wk of amiloride 10 mg od and chlorthalidone 25 mg od

### Outcome measures

The primary outcome measure is seated, office, unattended systolic blood pressure (SBP) measured 8 weeks after each treatment period begins. A validated, automated, brachial BP machine—the Omron HEM-907—will be used to record 3 serial measurements.

Secondary and tertiary outcome measures are shown in Table IV. Analysis of potential genetic polymorphisms associated with response to antihypertensive treatment will be limited to those polymorphisms previously linked to BP via genome-wide association studies.^14^ Preliminary retrospective analyses of randomized controlled trials evaluating the efficacy of antihypertensive drugs suggest that many of these variants are also linked to antihypertensive drug response with relatively large effect size; such effects may arise as a result of an overlap between the homeostatic pathways mediating BP control and drug targets. Additionally, genomic variants known to be specifically associated with antihypertensive drug response, published prior to the time of analysis, will be evaluated.^15^ This will maximize the power of our trial to detect genetic associations while avoiding the limitations of a candidate gene approach. The association with metabolites will be exploratory, using a “metabolite-wide” association study approach, while also revisiting metabolites that have previously been deemed significant in published metabolite-wide association studies at the time of analysis.16., 17.

**Table IV:** Outcome measures

Primary outcome measure
- Seated, automated, unattended, office SBP, measured approximately 8 wk after each treatment
Secondary outcome measures
- All participants
- Seated, automated, office DBP
- Detailed SDE
- Core cardiovascular measurements
- Body composition assessment to determine lean and fat tissue mass
- Pulse wave analysis (carotid & femoral arteries) to derive pulse wave velocity, central (aortic) waveforms, and central BP
- Echocardiography (including left ventricular mass and volume assessments)
- Dundee (3-min) step test (baseline only)
- Subgroups
- ABPM and/or HBPM
- Optional cardiovascular measurements
- Heart rate variability
- Regional arterial diameters
- cardiac output (CO) and stroke volume (SV) assessment (using a noninvasive, inert gas rebreathing technique)
Tertiary outcome measures
- Hemodynamic and genomic measures
- Baseline demographics comparison
- Urine drug screening (random subgroup sample)

SBP: systolic blood pressure; DBP: diastolic blood pressure; SDE: self defined ethnicity; ABPM: ambulatory blood pressure monitoring; HBPM: home blood pressure monitoring; CO: cardiac output; SV: stroke volume.

### Sample size calculation

To identify the effect of ethnicity upon response to the various treatment options trialed with 98% power, 200 patients from each of the 3 ethnic groups, that is, 600 subjects per trial arm, will be required. This assumes an SD for daytime systolic BP of 8 mm Hg, use of a global test of interaction at the 5% significance level, and a single interaction of 4 mm Hg with others of 0 mm Hg. Should the single interaction be 3 mm Hg, the power is reduced to 81.3%. However, 4 mm Hg is an effect size approximately 50% of that reported for some of the agents to be used in this trial when studied among black and white participants in the United States.^18^

To allow for a 10% dropout rate, the trial will enroll 660 participants per trial arm, with 220 subjects from each of the 3 ethnic groups. Recruitment for each ethnic group will cease when 220 participants are enrolled to ensure that equal numbers of patients are recruited. Subgroup assessments are not powered, as these are exploratory measures.

### Statistical methods

The 2 crossover trials will be considered distinct entities for analytical purposes; the results will be interpreted separately on an intention-to-treat basis. The primary end point—automated, office SBP—will be analyzed using a linear mixed-effects model. This end point will serve as the dependent variable, participant ID the random effect, with treatment factors, treatment period, ethnic group, and treatment by ethnic group the fixed effects. The global test of interaction, at a 5% significance level, will be used to determine whether the treatment effect varies with ethnicity. In the event of a significant global test, the efficacy of individual agents/treatments will be estimated with nominal 95% CIs; the assumptions of the model will be assessed using graphical methods, for example, a Q-Q plot and plots of residual versus fitted values. If any of the assumptions are violated, the dependent variable may be transformed to a normal distribution; if this fails to correct the distributional assumptions, nonparametric methods will be used.

Analysis of secondary and tertiary outcomes will be dependent upon the volume of data acquired; assessments of this (and the appropriate statistical methods) will be determined by the independent chair of the Trial Steering Committee (TSC) (see below). A detailed statistical analysis plan will be produced before the database is locked and/or before any interim analysis is performed.

### Interim analysis and sample size reassessment

With limited prior data describing intraindividual SDs in SBP, the multilevel nature of the trial design mandates an interim sample size reestimation. Statistically robust and confined to an analysis of SD in BP, this will be undertaken for each trial arm (monotherapy and dual therapy) after approximately 50 participants have completed at least 2 treatment periods. Given the likelihood that recruitment to the 2 arms will differ, it is anticipated that the sample size reestimations are unlikely to occur simultaneously.

Only results of the sample size reestimation will be communicated to investigators; the details of any treatment effects will not be made available.

### Organization and funding

The trial, sponsored by Cambridge University Hospitals NHS Foundation Trust and the University of Cambridge, is led by the Cambridge Clinical Trials Unit at Cambridge University Hospitals NHS Foundation Trust. Addenbrooke's Hospital, Cambridge, serves as the coordinating center, whereas the Cambridge South (East of England) Research Ethics Committee provided a favorable ethical opinion for the protocol in October 2016.

A TSC consisting of experienced clinical investigators provides overall supervision for the trial, ensuring that it is conducted in accordance with the protocol and Good Clinical Practice. Convening at regular intervals and independently chaired (Prof Peter Sever), the committee assumes overall responsibility for participant safety, consideration of new information, and reviewing data, as specified in the TSC charter.

Part of the wider AIM-HY consortium, the AIM-HY INFORM trial is funded by the Medical Research Council and British Heart Foundation. The sponsors and funding organizations have no role in the study design, study management, or data interpretation. The investigators (authors) alone are responsible for these aspects of the study, as well as any data analysis, the drafting and editing of manuscripts, and their final contents.

### Current status

At present, 8 investigation centers are actively recruiting patients, and it is anticipated that the trial will be completed by mid-late 2020; the results will be reported approximately 6-9 months later. The first patient was consented on 20 February 2017 and randomized on 6 March 2017; as of 15 May 2018, 318 patients had been consented, with 252 randomized. The study has been registered with the Web site ClinicalTrials.gov (NCT02847338).

## Commentary

Ethnicity influences BP status, at both an individual and population level; epidemiological data from the United States demonstrate a greater prevalence of hypertension among African Americans, along with poorer BP control among this cohort of the population.19., 20., 21., 22. However, data describing the prevalence of hypertension among the United Kingdom's various ethnic groups are inconsistent. Some UK studies describe a greater prevalence of hypertension, and significantly higher mean BP, in both Afro-Caribbean and South Asian populations when compared to the indigenous white population.23., 24. Other studies have shown significant variations in BP data among subgroups of the South Asian population, with some sections of this community reportedly having lower BP readings than white adults; at various times, these differences have been attributed to religion (eg, Muslim, Sikh, Hindu), nation of origin (eg, India, Pakistan, Bangladesh), and cultural grouping (eg, Gujarati, Punjabi).25., 26., 27. Furthermore, studies which consider admixed populations are sparse despite the fact that this cohort of the population is growing in size; more than 1 million people in the United Kingdom identify as “mixed,” with 0.8% of the English and Welsh population describing themselves as being “white-black Caribbean,” 0.6% “white-Asian,” 0.3% “white-black African,” and 0.5% “other mixed.”^8^

The pathophysiology of hypertension also appears to vary between ethnic groups. In the UK South Asian population, elevated sympathetic activity, arising from an increased prevalence of central obesity and insulin resistance, is believed to be causally related to BP, whereas “low-renin” hypertension is commonly described among black adults, with the resulting salt and water retention a significant determinant of BP status.28., 29., 30., 31., 32. In contrast, salt-sensitive hypertension is relatively infrequent in white adults.31., 32. Black patients are also reported to express variants of several genes, most frequently a threonine to methionine substitution, T594 M, affecting the renal tubular absorption of sodium and water.^33^ Phenotypically akin to Liddle syndrome, this pathophysiological mechanism is primarily mediated via the renal tubular epithelial sodium channel.^34^

Treatment on the basis of these observations has resulted in the use of diuretics in hypertensive adults with low plasma renin activity and antagonists of the renin-angiotensin axis (eg, angiotensin-converting enzyme inhibitors [ACEi], angiotensin II receptor blockers [ARBs], or β-blockers) in individuals with higher plasma renin activity.35., 36., 37., 38. In addition to this, the parallels to Liddle syndrome have led a number of authors to advocate the use of amiloride among cohorts of black patients, both in the UK and elsewhere, whereas the elevated sympathetic activity observed among South Asians has been used to justify ACEi, ARB, and β-blocker use in this group.28., 39.

Although data relating to Asians in the North American literature are limited—and where it is available, “Asian” frequently equates to “Far Eastern” (Chinese, Japanese, etc)40., 41.—results from several large US studies provide some evidence of ethnic variation in response to antihypertensive drugs.34., 42., 43., 44., 45., 46., 47. In broad terms, greater falls in BP are achieved with thiazide/thiazide-like diuretics in black subjects with hypertension when compared to their white peers; conversely, ACEi are less effective in this cohort. In the Antihypertensive and Lipid-Lowering Treatment to Prevent Heart Attack Trial, patients on chlorthalidone achieved better BP control than those receiving lisinopril or amlodipine. Those using lisinopril had a greater risk for stroke (risk ratio [RR]: 1.40, 95% CI: 1.17-1.68), combined cardiovascular disease (RR: 1.19, 95% CI: 1.09-1.30), and heart failure (RR: 1.30, 95% CI: 1.10-1.54) compared with those receiving chlorthalidone, treatment differences which were far more pronounced in African Americans when compared with whites.^42^ Furthermore, the Antihypertensive and Lipid-Lowering Treatment to Prevent Heart Attack Trial researchers reported a higher risk of stroke in African American hypertensive patients treated with lisinopril as opposed to amlodipine (RR: 1.51; 95% CI: 1.22-1.86), an association which was not observed in non–African Americans (RR: 1.07; 95% CI: 0.89-1.28).^42^ Subsequent meta-analyses reiterate these findings, demonstrating no evident benefit from ACEi in achieving diastolic blood pressure (DBP) goals for African American hypertensive patients.48., 49. Conversely, ACEi may offer substantial benefits for African Americans with hypertensive renal disease; in the African American Study of Kidney Disease and Hypertension, ramipril slowed renal disease progression in African Americans irrespective of whether proteinuria was present, more so than amlodipine or metoprolol.^50^ However, demographic differences with the United Kingdom (eg, a large South Asian population, population “admixture,” first/second-generation immigrants as opposed to a population resident for 2-3 centuries), along with variations in vascular risk, diet, and weight, restrict the utility of US data.

The evidence for differential responses to antihypertensive drugs on the basis of ethnicity in the United Kingdom is more limited, with an analysis from 203 African, 132 South Asian, and 4,368 white participants in the UK arm of the Anglo-Scandinavian Cardiac Outcomes Trial perhaps the most robust source of data.^51^ This sought to determine whether there were ethnic variations in response to monotherapy using β-blockers (atenolol) or calcium channel blockers (amlodipine) and then to add-on therapy with a thiazide diuretic (bendroflumethiazide) or ACEi (perindopril). The degree of BP reduction achieved in black patients receiving atenolol monotherapy was significantly lower when compared to white patients; South Asian patients achieved an intermediate treatment effect. Amlodipine monotherapy resulted in similar BP-lowering effects across all 3 ethnic groups, as did the addition of bendroflumethiazide to atenolol. However, the addition of perindopril to amlodipine resulted in statistically significant BP differences; white patients achieved a further 1.7–mm Hg fall in SBP (95% CI: −2.8 to −0.7 mm Hg), black patients exhibited a diminished response (SBP change: −0.8 mm Hg; 95% CI: −2.5 to +4.2 mm Hg), whereas South Asians demonstrated a greater response (SBP change: −6.2 mm Hg; 95% CI −10.2 to −2.2 mm Hg).^51^

The AIM-HY INFORM trial aims to optimize first- and second-line antihypertensive therapies for the multiethnic population of the United Kingdom. In doing so, it will refine the “black versus white” approach embodied in the current NICE guideline and further codify the treatment of hypertension. The systematic and robust trial data produced will also compare the value of SDE against genetically defined ancestry and metabolomics, informing future studies in low- and middle-income countries, where the utilization of existing generic drugs in a resource-efficient manner is imperative.
